# MAPKs in the early steps of senescence implemEMTation

**DOI:** 10.3389/fcell.2023.1083401

**Published:** 2023-03-16

**Authors:** Carlos Anerillas, Gisela Altés, Myriam Gorospe

**Affiliations:** Laboratory of Genetics and Genomics, National Institute on Aging Intramural Research Program, National Institutes of Health, Baltimore, MD, United States

**Keywords:** senescence, epithelial-mesenchymal transition (EMT), MAPKs, fibrosis, senescence-associated secretory phenotype

## Abstract

Evidence is accumulating that the earliest stages of the DNA damage response can direct cells toward senescence instead of other cell fates. In particular, tightly regulated signaling through Mitogen-Activated Protein Kinases (MAPKs) in early senescence can lead to a sustained pro-survival program and suppress a pro-apoptotic program. Importantly, an epithelial-to-mesenchymal Transition (EMT)-like program appears essential for preventing apoptosis and favoring senescence following DNA damage. In this review, we discuss how MAPKs might influence EMT features to promote a senescent phenotype that increases cell survival at the detriment of tissue function.

## Senescence and the SASP

Senescence is a cellular response to different types of sublethal damage characterized by indefinite cell cycle arrest and the secretion of bioactive molecules (Childs et al., 2014; Gorgoulis et al., 2019). This secretory trait, known as the SASP (senescence-associated secretory phenotype), has been proposed to be a core function of senescent cells, as it includes messengers and coordinators of the damage response within tissues like pro-inflammatory cytokines, growth factors, angiogenic factors, and matrix metalloproteases (Munoz-Espin et al., 2013; Storer et al., 2013; He and Sharpless, 2017; Antelo-Iglesias et al., 2021; Anerillas et al., 2022a). There is increasing evidence that cellular senescence is beneficial for organism homeostasis, mediating physiologic processes like embryonic development as well as tissue repair and tumor suppression. However, when senescent cells accumulate to aberrantly high levels, their normal homeostatic influence is disrupted, creating a pro-inflammatory state that impairs organ function, reduces regeneration, and fuels aging-associated decline and disease (Lopez-Otin et al., 2013; He and Sharpless, 2017). In this regard, senescent cells have been linked to many age-related diseases including neurodegeneration, diabetes, obesity, sarcopenia, and cardiovascular disorders (Chaib et al., 2022).

Therefore, careful regulation of the levels of senescence and SASP is necessary for maintaining organ homeostasis in response to tissue damage. Mostly through the SASP, senescent cells also help coordinate the response of nearby and distant damaged cells and even trigger secondary senescence (Acosta et al., 2013; Parry et al., 2018; Teo et al., 2019). However, persistent SASP can trigger an exacerbated response that hampers tissue regeneration through uncontrolled inflammation, sometimes reducing the stemness of key cells (Ritschka et al., 2017). In short, a balanced and transient SASP is believed to be beneficial, but prolonged SASP could be detrimental.

Besides circulating factors, senescent cells also produce extracellular matrix (ECM) structural proteins (Hoare et al., 2016). Although this trait is less well understood, it is increasingly recognized as being important for the overall senescence program, as highlighted in recent reports on the relevance of integrins for senescent cells (Blokland et al., 2020; Borghesan and O'Loghlen, 2017; Rapisarda et al., 2017; Jung et al., 2019; Shin et al., 2020; Anerillas et al., 2022b). Therefore, it is essential to understand the different stages and facets of the secretory phenotype in senescent cells, so as to gain a broader view of its diverse impact, particularly given its critical roles in tissue repair.

## Stages of senescence and the EMT

The harmful effect of senescent cells in the aforementioned age-related pathologies has stimulated an escalation of efforts to find therapeutic strategies that reduce senescent cells (Chaib et al., 2022). Several studies have shown that an efficient way to eliminate senescent cells is by forcing them into apoptosis, given that senescent cells manifest an underlying pro-apoptotic state elicited by the damage they have endured (Yosef et al., 2016; Baar et al., 2017; Anerillas et al., 2022b). Even though the response to damage is known to be a dynamic process, the specific stages during which molecular decisions are made to undergo senescence or apoptosis have not been thoroughly investigated. A deeper knowledge of these phases can help elucidate points of vulnerability for developing effective interventions to eliminate senescent cells. A recent report of early differences between cells committed to either senescence or apoptosis following DNA damage (Anerillas et al., 2022b) revealed that at the senescence-vs-apoptosis decision fork, cells committed to survival and senescence showed an epithelial-to-mesenchymal transition (EMT)-like transcriptomic program that appeared to be critical for cell viability. The EMT is a developmental process through which epithelial cells lose their polarity and acquire mesenchymal characteristics to form new tissue structures and often to improve cell survival (Tiwari et al., 2012; Dongre and Weinberg, 2019). In our recent studies, we found that senescence-committed cells displayed higher levels of activity of the mitogen-activated protein kinase (MAPK) p38 that appeared crucial for favoring survival over apoptosis. In this review, we discuss the current understanding of how MAPK activity may regulate the decision between senescence and apoptosis through the EMT.

## Cell senescence is a dynamic process

Following damage, cells discern at the molecular level whether to undergo rapid mending (for repairable damage), apoptotic death (for catastrophic damage), or sustained senescence (for sublethal damage) (Childs et al., 2014; Anerillas et al., 2022b). These programs reflect an evolutionary adaptation to damage that coordinates the responses to injury in multicellular organisms (van Deursen, 2014; Childs et al., 2015). In this regard, senescent cells serve as “sentinels” of the damage encountered, and they coordinate nearby and distant responses through the secreted factors acting in a paracrine and endocrine fashion to promote organism homeostasis (Munoz-Espin et al., 2013; Storer et al., 2013; Demaria et al., 2014). However, more comprehensive knowledge of these responses is needed, including the timeline of signaling events, the molecular factors produced, and the interaction of senescent cells with the environment and other cells.

Senescence is implemented in at least two distinct phases in fibroblasts (Hoare et al., 2016; Ito et al., 2017). During the earlier, pro-fibrotic stage, senescent cells produce extracellular matrix (ECM) proteins and general pro-EMT factors (mainly TGF-β) (Hoare et al., 2016; Sturmlechner et al., 2021). When this stage begins to decline, a more pro-inflammatory stage follows, during which cytokines and inflammatory mediators are robustly expressed, leading to enhanced immune surveillance and clearance of senescent cells (Tasdemir et al., 2016; Ovadya et al., 2018; Prata et al., 2018; Desdin-Mico et al., 2020). In fact, the dynamic change in the SASP is reminiscent of the overall process observed during wound healing (Rodrigues et al., 2019) and further supports the idea of senescent cells as vital elements of tissue remodeling after damage.

## Senescence or apoptosis? Messages from the EMT

The factors responsible for favoring a program of senescence over apoptosis following damage are not sufficiently known (Hoare et al., 2016; Lee and Schmitt, 2019; Anerillas et al., 2022b). We recently described how an EMT-like phenotype emerged as being critical for directing cells towards senescence instead of apoptosis, underscoring the value of this process (Anerillas et al., 2022b). Briefly, an EMT-like transcriptomic program, requiring integrin signaling, was found in cells that ultimately underwent senescence and not apoptosis. Moreover, the extracellular signal-regulated kinases 1 and 2 (ERK1/2) and c-Jun N-terminal kinase (JNK) were more active in cells committed to apoptosis, while p38 activation remained elevated in cells committed to senescence. We also previously associated pro-survival and EMT-like transcriptomic programs to the activity of MAPK ERK5 in cellular senescence (Anerillas et al., 2022a). However, the precise impact of MAPKs on the EMT program has not been fully explored in cellular senescence.

**FIGURE 1:**
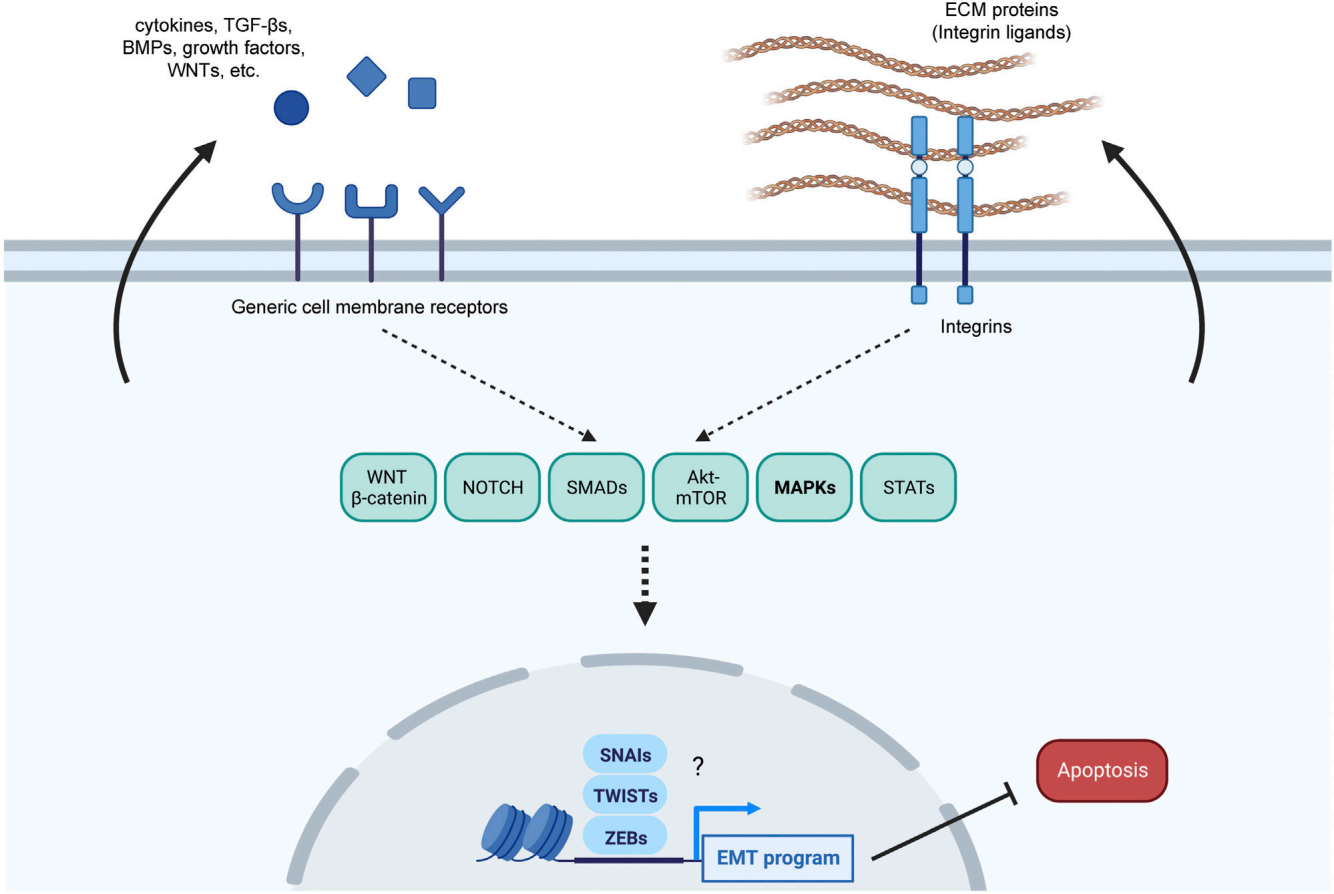
Overview of possible senescence-related pro-EMT pathways. Damaged cells might both respond to and produce pro-EMT factors to adjust their response to the extent of the damage suffered. Several pro-EMT factors such as secreted ligands (cytokines, TGF-βs, BMPs, growth factors, NOTCH ligands, WNTs) and ECM proteins that bind to integrins are produced by senescent cells and can further enhance senescence over apoptosis; the arrows arising from the cytosol towards the extracellular space represent possible autocrine loops. These ligands might be responsible for the modulation of several EMT-related pathways, including MAPKs, to implement an EMT program, which further relies on core pro-EMT transcription factors, including the SNAIs (SNAIL and SLUG), TWISTs (TWIST1 and TWIST2), and ZEBs (ZEB1 and ZEB2).

In this regard, the impact of MAPKs on the EMT in other systems could shed light on their impact on EMT in senescence. The EMT program is crucial for processes of tissue remodeling in embryonic development and wound healing (Dongre and Weinberg, 2019), both linked to cell senescence (Huang et al., 2022). Furthermore, many of the molecules secreted by senescent cells, including cytokines, growth factors, and ECM proteins, are known EMT triggers (Krtolica et al., 2001; Parrinello et al., 2005; Yilmaz and Christofori, 2009; Coppe et al., 2010; Acosta et al., 2013; Lamouille et al., 2014; Hoare et al., 2016; Cherubini et al., 2017; Liu et al., 2018; Scott et al., 2019; Hou et al., 2021; Anerillas et al., 2022a). Downstream of membrane receptors, many signaling pathways related to both EMT and senescence are activated along with the MAPKs; these signaling modules include WNT-β-Catenin, NOTCH, SMADs, AKT-mTOR, and STATs (Acosta et al., 2013; Munoz-Espin et al., 2013; Herranz et al., 2015; Laberge et al., 2015; Hoare et al., 2016; Bryson et al., 2017; Kandhaya-Pillai et al., 2017; Gibaja et al., 2019; Anerillas et al., 2020; Anerillas et al., 2022a). The same pathways in turn control the function of core EMT transcription factors SNAI1 (SNAIL), SNAI2 (SLUG), ZEBs (ZEB1 and ZEB2), TWISTs (TWIST1 and TWIST2), often activated to implement a pro-fibrotic extracellular matrix-remodeling state and inhibit apoptosis (Dongre and Weinberg, 2019) (Figure 1). While MAPKs are activated in both EMT and senescence, the specific ways how MAPKs control the EMT phenotype acquired in early stages of senescence are poorly understood. The pro-survival EMT phenotype seen in early senescence likely relies on the function of NOTCH and different MAPKs such as ERK1/2, ERK5, or p38, as they are key signaling nodes that are active at initial stages of senescence (Freund, Patil, and Campisi, 2011; Hoare et al., 2016; Anerillas et al., 2022a; Anerillas et al., 2022b). Notably, these same signaling factors regulate the function of the above-mentioned core EMT transcription factors that promote survival over apoptosis and can induce the expression of pro-fibrotic proteins (Lamouille et al., 2014; Dongre and Weinberg, 2019).

In turn, the molecules secreted by senescent cells may themselves reinforce the EMT program in an autocrine or paracrine manner, thus also promoting survival over apoptosis in response to injury. As mentioned above, many of the proteins secreted by senescent cells promote EMT in multiple paradigms (Coppe et al., 2010; Acosta et al., 2013; Hoare et al., 2016; Hou et al., 2021), and senescent cells express many of the receptors to which these extracellular ligands bind (Acosta et al., 2013; Rapisarda et al., 2017; Anerillas et al., 2022a; Anerillas et al., 2022b). Therefore, the senescence-vs-apoptosis balance could be regulated by the degree of commitment to an EMT-like program, possibly regulated in part by extracellular ligands, although this hypothesis awaits to be tested.

## MAPKs in senescent cell fate decisions

The MAPK superfamily of proteins comprises a large number of signaling kinases with three central pathways: ERKs (mainly ERK1/2 and ERK5), p38s, and JNKs (Cargnello and Roux, 2011; Anerillas et al., 2020); JNK and p38 are sometimes referred to as stress-activated protein kinases (SAPKs). These effector proteins share signaling inputs from upstream kinases and control cellular responses by phosphorylating downstream effectors, such as other kinases, transcription factors, and RNA-binding proteins. Most of our current knowledge linking MAPKs and the EMT is from cancer studies (Gui et al., 2012; Huang et al., 2016; Bhatt et al., 2021; Liu et al., 2022), generally describes MAPKs as promoting the EMT in response to mitogens and stress (Dongre and Weinberg, 2019), and point to significant crossregulation in the activation of core EMT transcription factors by different MAPKs (Hong et al., 2011). Despite our incomplete understanding of the role of MAPKs in controlling the EMT-like phenotype at early senescence, some details are emerging, as discussed below.

Treating embryos with the ERK1/2 inhibitor U0126 prevents cellular senescence in the apical ectodermal ridge, a transient embryonic structure essential for proper development (Storer et al., 2013), although ERK1/2 can also promote apoptosis directly when excessively activated (Sugiura et al., 2021) and indirectly by promoting SMAD activity (Hough et al., 2012; Shi et al., 2020). Thus, ERK1/2 could gradually transition from a pro-senescence (and therefore pro-survival) to a pro-apoptotic role according to the strength of its activation, as observed for p53 (Hafner et al., 2019). Importantly, the activity of ERK1/2 increases *via* ECM receptors and ECM ligands (Sheng et al., 2017; Ohkuro et al., 2018; Olea-Flores et al., 2019), and thus ERK1/2 activity could represent a key signaling node to adjust cell outcome to ECM status (Anerillas et al., 2020). The evidence that ERK1/2 activate EMT core transcription factors like SNAIL, SLUG, TWIST, ZEB1, and ZEB2 (Weiss et al., 2012; Strippoli et al., 2015; Chiu et al., 2017; Sinh et al., 2017; Yuan et al., 2022) is mainly obtained from cancer cell studies.

ERK5 has also been associated with the EMT program downstream of mitogenic factors, TGF-β, and integrins (Bhatt et al., 2021). Interestingly, ERK5 is critical for BDNF to enhance senescent cell viability (Anerillas et al., 2022a), and was proposed to enhance the EMT program (Jia et al., 2015; Cherubini et al., 2017; Tinaburri et al., 2021). Remarkably, ERK5 participates in an extracellular signaling program involving BDNF to sustain neuronal viability and promote neurogenesis (Liu et al., 2003; Wang et al., 2006), the latter linked to EMT (Singh et al., 2016). In cancer cells, ERK5 either promotes or inhibits SNAIL function (Marchetti et al., 2008; Xu et al., 2021), although ERK5 activation usually promotes EMT by increasing the function of SLUG, ZEB1, and ZEB2 (Arnoux et al., 2008; Antoon et al., 2013a). Thus, ERK5 may be a pivotal MAPK controlling an EMT-like phenotype in early senescence.

Similarly, p38 has been linked to the EMT, especially downstream of TGF-β (Yu et al., 2002; Zohn et al., 2006; Passos et al., 2010; Lamouille et al., 2014; Pang et al., 2016; Corre et al., 2017; Witte et al., 2017). p38 was also recently described as enhancing survival early in the senescence response by promoting EMT downstream of an integrin-FAK-SRC signaling program (Anerillas et al., 2022b). A key way how p38 promotes EMT in cancer cells is by stabilizing and increasing the function of some of the EMT core transcription factors such as SNAIL, ZEB1, and TWIST1 (Hong et al., 2011; Antoon et al., 2013b; Werden et al., 2016; Ryu et al., 2019).

Finally, JNKs have been proposed to control the progression of the EMT program downstream of TGF-β actions (Lamouille et al., 2014; Sahu et al., 2015). Some studies suggest that the JNK pathway promotes EMT by regulating the expression of transcription factors and signaling molecules involved in the EMT process. For instance, JNK activates the transcription factor AP-1, which can promote the expression of genes associated with EMT, such as SNAIL, SLUG, and TWISTs in both cancer and normal cells (Li et al., 2010; An et al., 2013; Feldker et al., 2020).

**FIGURE 2:**
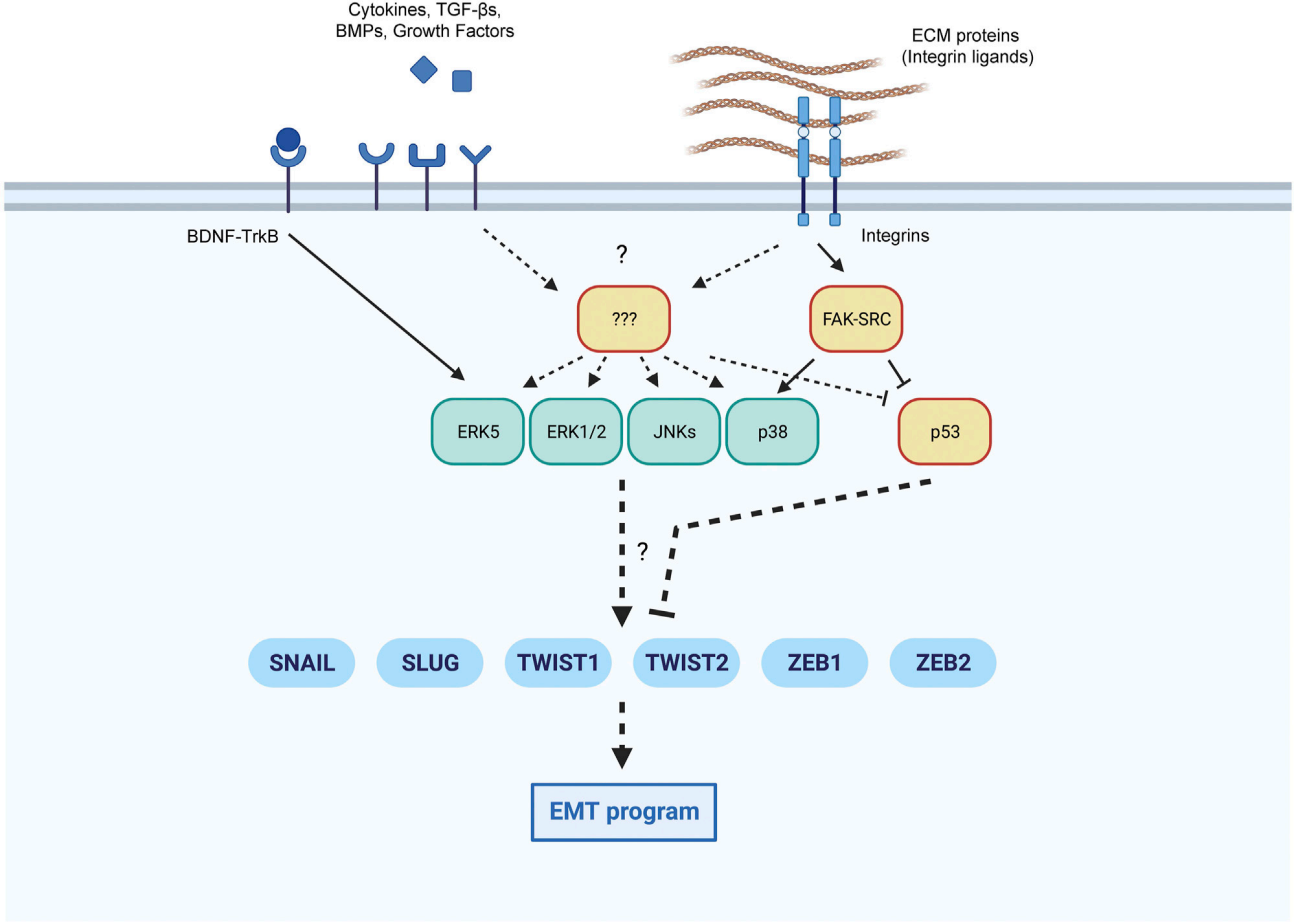
Proposed coordination of EMT core transcription factors by MAPKs and p53. Extracellular inputs can modulate the implementation of an EMT program in conditions favorable to senescence. We highlight the pathways leading to the activation of EMT transcription factors in senescence by MAPKs and p53, with known pathways indicated with continuous lines, and additional possible pathways with discontinuous lines.

In summary, the ability of MAPKs to drive a cellular outcome like senescence, appropriate to the type and extent of damage endured, might depend, at least in part, on their ability to implement an early EMT phenotype. Nonetheless, the EMT is a complex, cell type-dependent program that relies on multiple transcription factors, sometimes shared and sometimes exclusive to each cell context (Zavadil et al., 2004; Leroy and Mostov, 2007; Dhasarathy et al., 2011; Ganesan et al., 2016; Dongre and Weinberg, 2019; Sundararajan et al., 2019; Brabletz et al., 2021). Therefore, the cell-specific modulation of EMT core factors by the different MAPKs could explain our own findings that activation of p38 and ERK5 promotes EMT, while ERK1/2 and JNKs do not (Figure 2), although studies in other senescence paradigms are needed.

## Opposite influence of the EMT and p53 programs in the survival-death balance of senescent cells

It was proposed that proteins like TWIST and ZEB1 from the EMT program opposed senescence by inhibiting p53 (Thiery et al., 2009; Smit and Peeper, 2010). This antagonism is in keeping with the existence of a balance between competing signaling pathways in other aspects in senescence (Coppe et al., 2008; Hoare et al., 2016). Some level of restraint of p53 activity by EMT would enable cell viability while arresting proliferation, since p53 is essential for both senescence and apoptosis (Childs et al., 2014; Hafner et al., 2019). At the same time, the EMT program could also favor viability by promoting the increase of pro-survival proteins, as seen in cancer (Barrallo-Gimeno and Nieto, 2005; Lamouille et al., 2014; Gupta and Maitra, 2016), although these possibilities need to be investigated in senescent cells.

Conversely, p53 can inhibit EMT by controlling several main signaling pathways implicated in EMT implementation. For instance, forcing an increase in p53 levels at early senescence triggered by combined treatment with the genotoxic agent etoposide and the p53 activator Nutlin3a reduced STAT3 activation and lowered some EMT markers (Anerillas et al., 2022a). At the same time, p53 is required for STAT3 activation, indicating that p53 might serve as a molecular rheostat that promotes STAT3 activity and EMT when moderately active, but suppresses STAT3 and EMT when excessively active. p53 can affect other EMT-related pathways in different and complex ways; for example, the WNT-β-Catenin pathway is repressed by the p53-induced microRNA miR-34 (Kim et al., 2011), and similar to the effect on STAT3, p53 can both inhibit and be required for NOTCH expression in different cell contexts (Lefort et al., 2007).

The regulation of MAPKs by p53 in this paradigm is likely complex as well. p53 can inhibit MAPK activity (e.g., by inducing phosphatases like the dual-specificity phosphatases, DUSPs), but the specific MAPKs inhibited vary depending on the cell type, the stimulus, and the phosphatases expressed (Lu et al., 2007; Yu et al., 2007; Lokareddy et al., 2013; Fischer, 2017). For example, a p53-mediated rise in the levels of the phosphatase WIP1 suppressed p38 activation during senescence (Freund et al., 2011), although WIP1 suppresses other MAPKs in different cell types (Goloudina et al., 2016). DUSP14, another p53-regulated MAPK phosphatase (Fischer, 2017), differentially inhibits ERK1/2, p38, and JNKs depending on the cell context (Chen et al., 2019). At the same time, p53 also favors the activation of certain MAPKs by transcriptionally inducing the expression of EPHA2 (Macrae et al., 2005) or GADD45A (Salvador et al., 2013), which trigger the activation of ERK1/2 and p38, respectively. In short, the influence of p53 on MAPKs during early senescence is complex, depends on cell context, and is likely interdependent on other signaling events, as reported in other systems (Cargnello and Roux, 2011; El-Brolosy and Stainier, 2017).

The SMAD-dependent TGF-β and BMP pathways are also influenced by p53. TGF-β and p53 induce p21 expression (Dupont et al., 2004; Elston and Inman, 2012; Baar et al., 2017), and may jointly support the implementation of an EMT-like program in senescent cells (Cordenonsi et al., 2003; Teodoro et al., 2006; Sasai et al., 2008; Overstreet et al., 2014; Kawarada et al., 2016). Furthermore, p53 may affect the activity of SMADs (Chang et al., 2016) by transcriptionally inducing the production of GDF15 (Zi et al., 2012) and LIF (Massague and Wotton, 2000; Fischer, 2017). On the other hand, excess p53 activity can inhibit the function of the TGF-β-SMAD2/3 axis by increasing production of COL1A2, a pro-fibrotic collagen (Ghosh et al., 2004), or by tilting the balance between SMAD2/3 and SMAD1/5/9 (Guo and Wang, 2009; Zou et al., 2021) to affect cell outcomes. As TGF-β can ensure viability or promote apoptosis depending on the cell status (Zhang et al., 2017), it will be critical to understand whether p53 somehow controls the production of proteins in the TGF-β family during cell fate decisions to alter the balance between senescence and apoptosis.

Finally, p53 opposes EMT by inhibiting SLUG *in vivo* (Rinon et al., 2011; Jackson-Weaver et al., 2020) and TWIST1 and ZEB1 in cancer cells (Chang et al., 2011; Yang-Hartwich et al., 2019; Semenov et al., 2022). Despite ample evidence that coordinated p53 and EMT programs critically determine cell fate after injury, these roles have not been thoroughly elucidated in cell senescence.

## Response to EMT-activating factors and their links to fibrosis

**FIGURE 3:**
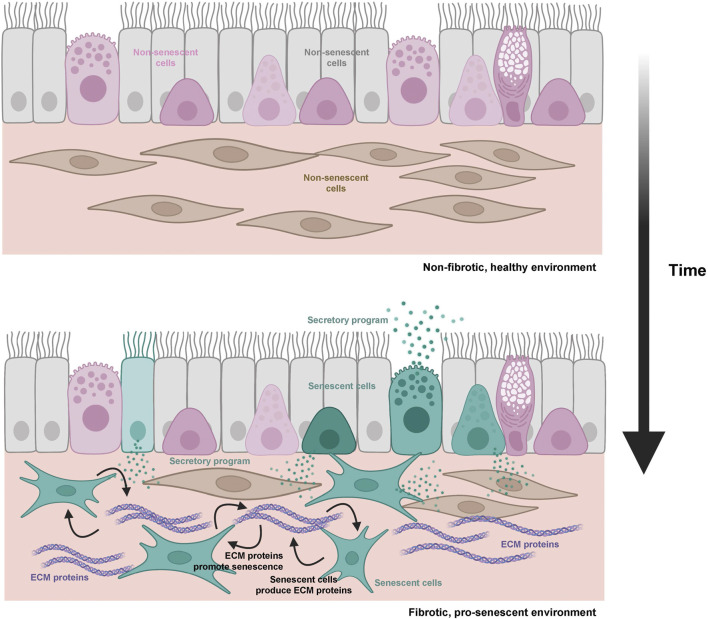
Schematic representation of the hypothesis connecting aging, fibrosis, and cellular senescence. During aging, accumulating senescent cells (teal) and ECM pro-fibrotic proteins (purple) can engage in a cycle in which both processes are reciprocally stimulated (regulatory loops are represented by black arrows).

Although it has not been studied in depth in tissues and organs, cultured senescent cells produce ligands that promote EMT, such as TGF-β and ECM matrix proteins (Acosta et al., 2013; Hoare et al., 2016). Moreover, activation of integrin signaling inhibits apoptosis by implementing an EMT-like program, both in cultured cells and in live animal models (Anerillas et al., 2022b), and integrin signaling is important for promoting cellular senescence (Borghesan and O'Loghlen, 2017; Rapisarda et al., 2017; Jung et al., 2019; Shin et al., 2020). Therefore, these and other signaling factors related to EMT implementation could be crucial in determining cell fate in the organism. Senescent cells present in tissues undergoing regeneration could both respond to and produce pro-fibrotic proteins, and thereby possibly exacerbate fibrosis (Schafer et al., 2017; Blokland et al., 2020; Levi et al., 2020; Hernandez-Gonzalez et al., 2021). This idea is supported by findings such as the pro-fibrotic early stages of senescence, characterized by TGF-β release and ECM protein deposition (Hoare et al., 2016), and the ability of SRC-FAK signaling to promote survival and senescence in pro-apoptotic conditions (Anerillas et al., 2022b). The latter finding suggests that cell fate can be driven by mechanisms external to the cell where the actual DNA damage occurs. In other words, signaling controlled by extracellular proteins could serve to warn surrounding cells of local damage. In agreement with this influence, fibrotic tissues could reciprocally enhance the accumulation of senescent cells by favoring the survival of damaged cells through pro-EMT signaling. This positive feedback might help explain the decreased regenerative capacity of several organisms with aging (Figure 3).

The connection between EMT and fibrosis is well established (Ovadya and Krizhanovsky, 2015; Di Gregorio et al., 2020), and the conversion of parenchymal cells into fibroblasts capable of producing ECM proteins is characteristic of declining tissue fitness. Therefore, an increase in the proportion of parenchymal cells undergoing EMT in tissues leads to fibrosis and loss of homeostasis. The fact that senescent cells produce pro-EMT proteins suggests that they may contribute to fibrosis in detrimental scenarios (Schafer et al., 2017; Levi et al., 2020; Ritschka et al., 2020; Anerillas et al., 2022a). Consequently, increased signaling to induce EMT *in vivo* could further favor senescence over apoptosis of damaged cells; this process would enable tissue repair, but it would also promote fibrosis when senescent cells accumulate in excess.

## Concluding remarks

A strong connection is emerging between cellular senescence and EMT, two processes associated with fibrosis (Ovadya and Krizhanovsky, 2015; Schafer et al., 2017; Di Gregorio et al., 2020; Levi et al., 2020; Ritschka et al., 2020; Anerillas et al., 2022a). It is likely that features connecting senescent cells to integrin signaling and the ECM have not been fully recognized until now. For example, the enlarged and flattened morphology of senescent cells, better appreciated in culture, likely reflects focal adhesion processes (Bauer et al., 2019) in addition to hyperactivated mTOR signaling, as previously proposed (Demidenko and Blagosklonny, 2009).

In conclusion, a fuller characterization of pro-fibrotic ECM deposition, cell senescence, and the role of ECM proteins supporting both processes is warranted. This knowledge will accelerate the identification of therapeutic strategies to intervene in fibrosis, tissue repair, aging, and age-associated disease.

## Data Availability

The original contributions presented in the study are included in the article/Supplementary Material, further inquiries can be directed to the corresponding author.
